# Altered IRE1α-Dependent Decidual Programming Links Impaired Inflammation and Angiogenesis in Recurrent Implantation Failure

**DOI:** 10.3390/biology15191693

**Published:** 2026-09-23

**Authors:** Lara Castagnola, Lourdes Materazzi, Daniel Paparini, Ana Schafir, Marcela Irigoyen, Esteban Grasso, Nancy Charó, Daiana Vota, Agustina Marcial, Diego Gnocchi, Alejandro Gustavo Martínez, Antonio Cattaneo, Lautaro Tessari, Vanesa Hauk, Soledad Gori, Rosanna Ramhorst

**Affiliations:** 1 Laboratorio de Inmunofarmacología, Instituto de Química Biológica de la Facultad de Ciencias Exactas y Naturales (IQUIBICEN), CONICET, Universidad de Buenos Aires, Buenos Aires C1428EGA, Argentina; 2 Fertilis Medicina Reproductiva, Buenos Aires B1609JEC, Argentina; 3 Instituto de Medicina Experimental (IMEX), Buenos Aires C1425AUM, Argentina

**Keywords:** recurrent implantation failures, decidualization, implantation, ER stress, inflammation, angiogenesis

## Abstract

Background: Decidualization alters the endometrial stromal cells (EnSC) secretome, allowing sterile inflammation associated with embryo implantation, but mechanisms that lead to alterations in this process are still unknown. We evaluated whether impaired IRE1α-mediated endoplasmic reticulum (ER) stress signalling contributes to defective angiogenic decidual programming in patients with recurrent implantation failure (RIF). Methods: Candidate pathways were identified with bioinformatic analyses of endometrial arrays and validated in endometrial biopsies from fertile and RIF women (secretory phase) by RT-qPCR and in isolated EnSC by flow cytometry. Functional alterations were evaluated using trophoblast spheroid and endothelial cell migration assays. ER stress was pharmacologically induced in EnSC using thapsigargin. Results: RIF endometria displayed impaired in vivo decidualization, with reduced expression of *IGFBP1* and *PGR*, together with altered expression of molecules involved in blastocyst adhesion. Conditioned media from RIF-derived EnSC-impaired trophoblast outgrowth. RIF samples displayed an anti-angiogenic and altered inflammatory profile, characterized by reduced intracellular IL-1β accumulation, decreased *VEGFA*, and increased *sFlt1* expression. Bioinformatic analyses identified IRE1α pathway as a central altered network associated with angiogenesis. Consistently, RIF samples showed reduced expression of *IRE1α*, *sXBP1*, and *NLRP3*. Moreover, ER stress induction in EnSC promoted endothelial cell migration. Conclusions: RIF is associated with defective angiogenic decidual programming linked to impaired IRE1α-mediated ER stress signalling. These findings identify the IRE1α axis as a potential pathway of interest for future therapeutic exploration in recurrent implantation failure.

## Simple Summary

Successful embryo implantation requires the coordinated regulation of embryo adhesion, inflammation, and blood vessel formation, but mechanisms that lead to alterations in these processes are still unknown. Even with a competent embryo, patients with recurrent implantation failure fail to achieve a successful pregnancy. By analyzing endometrial samples (tissue from the inner wall of the uterus) from fertile women and those with implantation failure, we found that patients who struggle to get pregnant have a defect in a key cellular stress pathway. This defect disrupts the necessary inflammation and blood vessel formation, making the endometrium less receptive to the embryo. Furthermore, when we artificially activated this stress response in endometrial cells from patients with recurrent implantation failure, we were able to partially restore their ability to promote blood vessel formation. We conclude that this cellular stress pathway contributes to preparing the endometrium for embryo implantation. Understanding this mechanism offers a promising new direction for investigating the mechanisms underlying implantation failure.

## 1. Introduction

Decidualization is an essential cellular process for embryo implantation and subsequent establishment of pregnancy [1]. During the secretory phase of the menstrual cycle, endometrial stromal cells (EnSC) undergo coordinated phenotypic and functional changes in response to hormonal and local mediators, generating a receptive uterine microenvironment capable of supporting embryo implantation [2]. The decidualization process is also characterized by profound changes in the secretory profile of EnSC, contributing to embryo–endometrial communication and the establishment of the maternal–foetal interface [3].

Successful embryo implantation requires the coordinated regulation of adhesion, inflammation, and angiogenesis. During the peri-implantation period, the endometrium develops a transient sterile inflammatory response characterized by the production of cytokines, prostaglandins, and other mediators involved in blastocyst attachment and trophoblast invasion [4]. Among these mediators, interleukin-1 beta (IL-1β), a classical inflammatory cytokine, is highly preserved in primates and is considered necessary for endometrial receptivity [5,6,7]. Currently, the evidence indicates that IL-1β contributes to decidualization by several mechanisms, such as integrin induction and reorganization of the cytoskeleton, to stabilize blastocyst adhesion by regulating trophoblast differentiation and its migratory and invasive capacity [8,9].

In parallel, embryo implantation is also accompanied by an angiogenic response, which sustains the eventual replacement of the endothelial cells by endovascular trophoblast cells, leading to the remodelling of spiral arteries. Therefore, a tightly regulated balance between pro- and anti-angiogenic mediators is critical to reach a successful implantation and placental development [10]. Increasing evidence indicates that both inflammatory and angiogenic responses associated with decidualization can be triggered by endoplasmic reticulum (ER) stress and the consequent unfolded protein response (UPR). In fact, activation of ER stress pathways during decidualization contributes to the acquisition of the decidual secretory phenotype and promotes the production of mediators involved in implantation and immune regulation [3,11,12].

Among the main sensors of the UPR, the inositol-requiring enzyme 1 alpha (IRE1α) pathway has emerged as crucial to promote angiogenesis and vascular adaptation [13]. Upon activation by ER stress, IRE1α utilizes its endoribonuclease activity to splice the mRNA of the X-box binding protein 1 (XBP1), resulting in the highly active transcription factor spliced XBP1 (sXBP1) [14]. Recent evidence demonstrates that the IRE1α-XBP1 signalling axis promotes angiogenesis by regulating the expression and intracellular stabilization of the vascular endothelial growth factor (VEGF) [13,15]. Interestingly, VEGF itself can selectively activate the IRE1α-XBP1 splicing pathway, suggesting a dynamic feedback loop that regulates endothelial cell proliferation and migration during vascular morphogenesis [16]. Collectively, these findings suggest that proper activation of the IRE1α pathway may be required to sustain the angiogenic decidual response necessary for embryo implantation.

Defective decidualization has been increasingly associated with reproductive disorders, including recurrent implantation failures (RIF), recurrent pregnancy losses, or complications associated with inadequate placentation, such as preeclampsia [17]. Particularly, patients with RIF are defined as women with a history of two or more cycles of in vitro fertilization (IVF) followed by embryo transfer, taking into consideration the quality of the transferred embryo and the patient’s age [18]. Although its aetiology is considered multifactorial, alterations in endometrial receptivity, stromal differentiation, and immune and angiogenic pathways have been proposed as central contributors to implantation failure [19]. Consistent with this, previous studies demonstrated that EnSC from RIF patients develop a deficient decidual response when differentiated in vitro [20]. However, the molecular mechanisms linking defective decidualization with impaired inflammatory and angiogenic responses in RIF remain poorly understood.

Based on this evidence, we hypothesized that defective IRE1α-mediated ER stress signalling contributes to impaired angiogenic decidual programming in RIF patients. To address this question, we combined integrated bioinformatic analyses, endometrial biopsies, and primary EnSC cultures from fertile women and RIF patients to investigate the relationship between ER stress, inflammation, and angiogenesis during embryo implantation. Furthermore, we explored whether pharmacological induction of ER stress could restore angiogenic response in EnSC derived from RIF patients.

## 2. Materials and Methods

### 2.1. Patients and Endometrial Biopsies

All the recruited patients who donated endometrial biopsies used in this research gave their informed consent. This project has the approval of the San Isidro Ethics Committee Board from Buenos Aires.

Fertile women (n = 12) were defined as non-pregnant women with a history of two or more normal pregnancies without any miscarriages, whereas RIF patients (n = 20) were defined as women with a history of two or more failed cycles of IVF followed by embryo transfer, without prior pregnancies. Transfers involved one euploid blastocyst-stage embryo (confirmed by preimplantation genetic testing for aneuploidies (PGT-A)) [18]. Demographic characteristics for both groups of women are detailed in Table 1. No significant differences in age and body mass index (BMI) were found between both groups.

Inclusion criteria for both groups were as follows: age from 30 to 46 years old, regular ovulatory cycles (28–31 days), normal uterine cavity evaluated by transvaginal ultrasound, and thyroid-stimulating hormone lower than 2.5 IU. Patients with infectious, endocrine, autoimmune or anatomic disease, endometriosis, adenomyosis, alterations in the uterine cavity, cancer, or abnormal levels of luteinizing hormone (LH) or follicle-stimulating hormone were excluded.

Endometrial biopsies were obtained in the secretory phase of the menstrual cycle, 6 days after the endogenous LH surge, which was measured with urine ovulation dipsticks (One+Test MedNet GmbH, Münster, Germany, sensitivity to LH > 20 mIU/mL) or serum LH > 15 mIU/mL. To confirm that patients were in the secretory phase of the menstrual cycle, progesterone levels were measured in peripheral blood (>3 ng/mL) at the time of sampling. Endometrial samples were obtained with Novak curette. Samples were stored at −20 °C in TriReagent until processed for genetic expression assays or transferred into tubes with 1ml DMEM-F12 media and stored at 4 °C for primary cultures.

### 2.2. RNA Isolation and Real Time PCR (RT-qPCR)

Total RNA was extracted using TriReagent according to the manufacturer’s instructions. A total of 1 μg RNA was reverse transcribed using MMLV reverse transcriptase, RNAse inhibitor, and oligo (dT) kit, and the cDNA was amplified using FastStart Universal SYBR Green PCR Master Mix (Cat. 4913850001, Roche, Sigma, São Paulo, Brazil) with specific primers. Primer sequences are shown in Table 2. Gene expression was quantified relative to the mRNA expression of the endogenous reference genes *GAPDH* or *YWHAZ* by comparative Ct method, using the 2^−ΔCt^ calculation and, in some cases, expressed as fold increase.

### 2.3. Endometrial Stromal Cells (EnSC) Isolation

Endometrial biopsies were used for EnSC isolation, as described before [21]. EnSC were centrifuged for 10 min at 210× *g* and resuspended with DMEM-F12 medium supplemented with 2 µg/mL insulin, 10% foetal bovine serum (FBS), 50 U/mL penicillin, 50 streptomycin µg/mL, and 2 mM glutamine to culture 50,000 cells/well in a 24-well plate. Cells were used immediately for flow cytometry analysis or cultured and expanded to develop primary cultures.

### 2.4. Flow Cytometry Analysis

The isolated cells were treated with brefeldin A (Cat. 420601, Biolegend, San Diego, CA, USA) for 2 h and then recovered by treatment with trypsin and centrifuged at 210× *g* for 5 min to perform intracellular staining of IL-1β (BD Biosciences, Heidelberg, Germany, Cat. 340516) and vimentin (Cat. 12-7018-81, eBioscience, San Diego, CA, USA) or the corresponding isotype controls using Intracellular Fixation and Permeabilization Buffer Set (Cat. 88-8824-00, Invitrogen, Waltham, MA, USA) as manufacturer’s protocol. In all cases, the stained cells were acquired using a FACSAria II Flow Cytometer (BD Biosciences), and the results of at least 20,000 events were analysed using FlowJo^®^ version 10.8.1 software.

### 2.5. In Vitro Decidualization Model

The immortalized human endometrial stromal cell (HESC) line was kindly provided by Dr. Gil Mor, Medical School, Wayne University, Detroit, MI, USA. Cells were routinely cultured in 24-well plates until reaching 80% confluence using HESC complete medium (DMEM-F12 with 10% FBS, 50 U/mL penicillin, 50 μg/mL streptomycin, and 2 mM glutamine). To ensure experimental integrity, HESC cultures were routinely tested for mycoplasma contamination using PCR-based assays and consistently tested negative. Cellular identity and integrity were continuously authenticated throughout the experiments by monitoring their characteristic spindle-shaped stromal morphology and their well-established functional capacity to decidualize in response to hormonal stimuli.

To induce differentiation into decidualized cells (Dec), HESC were stimulated with 2.5 mM dibutyryl-cAMP (db-cAMP) and 10 mM medroxyprogesterone acetate (MPA) for 8 days, replacing half of the culture media every 48 h, as previously described [22]. The efficiency of decidualization in this standardized model has been extensively validated in our laboratory through the evaluation of classical decidualization markers and cell viability [22]. After the eighth day, culture media were replaced and left for 48 h, and conditioned media was obtained and then used as control in the migration assay.

### 2.6. Evaluation of Trophoblast Migration by Blastocyst-like Spheroid Model

To evaluate the impact of RIF EnSC secreted factors on trophoblast migration, we used a blastocyst-like spheroid (BLS) in vitro model described previously by [23]. BLS were cultured for 48 h with conditioned media from HESC Dec (control) or conditioned media from primary cultures of RIF EnSC (RIF). Photographs were taken every 12 h with an Olympus BX61 (Olympus, Centre Valley, PA, USA). BLS area (spheroid plus outgrown migration area) was determined as the visible border of the outer cells of the migrating front using Fiji 2.9.0 ImageJ software (NIH, USA), and migration index was quantified as fold change relative to t0.

### 2.7. In Vitro Model of Implantation

Trophoblast cells from a confluent T25 flask of Swan-71 cells were trypsinized and plated into low-attachment P60 plates (Corning Incorporated, Corning, NY, USA). Formation of BLS was monitored until they reached a compact spherical morphology (48 h). Cell viability of the spheroids was evaluated by trypan blue staining (viability > 99%). Simultaneously, HESC cells decidualized, and non-decidualized were grown to confluence in 24-well plates (Greiner Bio-One, Kremsmünster, Austria). The inhibitor STF-083010 was added for 4 h prior to the invasion assay. Swan-71 spheroids were morphologically selected and stained with CFSE (CellTrace CFSE Kit, ThermoFisher Scientific, Waltham, MA, USA) according to provider’s instructions, and 10 spheroids per well were transferred using a transfer pipette and a dissecting microscope. The co-culture was maintained with DMEM supplemented with 10% FBS without differentiation stimuli or STF-083010. All co-cultures were monitored using a fluorescence microscope (Olympus Lifesciences, Centre Valley, PA, USA), and microphotographs were taken at 48 h [22]. Invasion index was analyzed as morphological change and calculated as “1-minor_axis/mayor_axis” of an ellipse surrounding the BLS, as previously reported using ImageJ software (NIH, USA) [22]. For each assay, the average of the invasion index of all the BLS on each well was considered as one single sample and used for the statistical analysis.

### 2.8. HUVEC Culture and Migration Assay

Human umbilical vein endothelial cells (HUVEC) were isolated from umbilical cord collected after normal full-term deliveries as described previously [24]. Angiogenic factors production by RIF EnSC was analysed by HUVEC’s wound healing assay as previously described [25,26]. During the 8-hour wound healing assay, cells were cultured with conditioned media from primary cultures of RIF EnSC previously treated with the potent ER stress inducer, thapsigargin (Tg, 1 µg/mL for 4 h) (RIF + Tg), or untreated (RIF). HUVEC migration arbitrary units (A.U.) were obtained by analysing the whole wound area, relative to t0, with ImageJ software.

### 2.9. Integrated Bioinformatics Analysis

A search of the possible signalling pathways involved in the processes of angiogenesis, ER stress, adhesion, and inflammation was carried out in public databases (Reactome, Gene Ontology, Wikipathways, and KEGG). From this search, 48 pathways were obtained, which were associated with each of the previously indicated processes. Subsequently, the genes that were part of the pathways were extracted, maintaining their association with each process, and duplicates were eliminated. Then, using public data arrays (GSE234354), we identified genes that were modulated in the secretory phase of the menstrual cycle in comparison with the proliferative phase in a healthy endometrium (|Fc| > 2; *p* < 0.05; false discovery rate (FDR) < 0.25). These thresholds were selected to stringently capture genes with a robust magnitude of change (|Fc| > 2) while maintaining an exploratory FDR cutoff suitable for heterogeneous human clinical samples, minimizing false negatives in complex biological networks. From these data, node tables (837 genes + 4 processes) were generated, and a network was built using Gephi 0.11.3 software. The shape of the network was obtained by the ForcedAtlas2 algorithm from random distribution until self-stabilization (using standard configuration parameters such as Scaling: 2.0, Gravity: 1.0, Tolerance: 1.0). It is important to note that the spatial layout, the relative distance between genes, and the node sizes were generated solely for visualization and clustering purposes and do not carry intrinsic biological relevance.

On the second stage, an endometrial biopsy array from fertile and RIF women (GSE92324) was selected. Genes that were significantly modulated (|Fc| > 2; *p* < 0.05; FDR < 0.25) were identified using the genepattern platform, and their modulation was plotted with different colours on the network created in Gephi. Finally, interactions between genes in the ER stress and angiogenesis processes were analysed using String 12.0.

### 2.10. Statistical Analysis

Statistical analysis was performed using GraphPad Prism 9 software (GraphPad Software Inc., San Diego, CA, USA). Normality of the data distribution was formally assessed using the Shapiro–Wilk test. Because the data did not pass the normality test, which is an expected outcome given the relatively small sample sizes, non-parametric tests were employed. For comparisons between two independent groups (e.g., endometrial biopsies or primary cells derived from distinct Fertile vs. RIF donors), the non-parametric Mann–Whitney U test was used. For paired experimental designs (e.g., primary EnSC from the same RIF donor cultured in the presence or absence of thapsigargin), the non-parametric Wilcoxon matched-pairs signed rank test was applied. For all functional in vitro assays, the sample size (n) represents independent biological replicates (i.e., primary cultures derived from distinct individual donors) and not technical replicates. Exact *p*-values are reported in each figure, and a *p*-value < 0.05 was considered statistically significant.

## 3. Results

### 3.1. Interconnection of Processes Associated with Embryo Implantation in RIF Patients

Given that endometrial receptivity may be compromised by alterations in the decidualization program, leading to further alterations in the accompanying inflammatory and angiogenic responses, impacting embryo implantation, we wondered if these alterations could be found in the endometria of patients with RIF. To address this broad question, an integrated bioinformatic analysis of endometrial biopsy arrays was performed. We searched for signalling pathways involved in the processes of ER stress, angiogenesis, adhesion, and inflammation in public databases, as described in the Section 2. We selected the genes that are modulated during the secretory phase of the menstrual cycle in comparison with the proliferative phase in healthy endometria. A network was generated using the Gephi program with the processes as nodes represented in green and the genes as pink dots (Figure 1), yielding a first network of 837 genes.

In a second step, we compared endometrial gene expression between fertile women and RIF patients using a public dataset and identified 144 differentially modulated genes, indicated in red (upregulated) and blue (downregulated) in Figure 1 (Figure 1, Table S1).

This analysis revealed that ER stress and angiogenesis process nodes shared a high proportion of dysregulated genes, with IRE1α emerging as a central connecting element between both processes. Based on this finding, a focused list of candidate genes involved in the processes of angiogenesis, ER stress adhesion, and inflammation was selected for experimental validation in endometrial biopsies from fertile women and RIF patients by RT-qPCR.

### 3.2. RIF Patients Display Alterations in the Decidualization Program

To characterize the alterations in the in vivo decidualization program suggested by the bioinformatic analysis, we evaluated the expression of key markers of decidualization in endometrial biopsies from RIF patients and fertile women obtained during the secretory phase of the menstrual cycle.

As shown in Figure 2A, the endometrial samples obtained from RIF patients showed a decreased expression of IGFBP1 (insulin-like growth factor-binding protein 1), a characteristic decidualization marker, as well as a decrease in the progesterone receptor (PGR), one of the main hormones involved in the decidualization program, compared to levels observed in fertile women (Figure 2B).

### 3.3. Secretome from Endometrial Stromal Cells from RIF Patients Impair Trophoblastic Migration

Considering the alterations in the decidualization markers, we next evaluated genes associated with the apposition and attachment of the blastocyst. We observed increased expression of mucin 1 (MUC1), a molecule that prevents blastocyst adhesion, and a decrease of the molecule involved in blastocyst attachment, the integrin subunit alpha 8 (ITGA8) in endometrial samples from RIF patients in comparison to fertile women (Figure 3A,B). These changes suggest an endometrial environment less permissive to blastocyst adhesion.

We then wondered how these alterations in decidualization and apposition could have effects in the secretome of EnSC that would impact the trophoblastic migration needed for embryo implantation. Using blastocyst-like spheroids (BLS), we performed a migration assay. We observed that conditioned media from EnSC isolated from endometrial samples from RIF patients reduced trophoblast migration and also displayed an altered trophoblast outgrowth (a rougher perimeter border) compared to conditioned media from in vitro decidualized cells (control) (Figure 3C,E). We detected a tendency to migrate less at 36 h, which became significant at 48 h (Figure 3D). Figure 3E shows a representative microphotography showing the altered growth pattern at 48 h of culture.

Together, these results suggest that the endometria from RIF patients display alterations in the decidualization program that would lead to an inadequate expression of characteristic markers associated with attachment and adhesion of the blastocyst and that these molecular alterations might be reflected functionally in impaired trophoblast migration.

### 3.4. RIF Patients Display Alterations in the Endometrial Inflammatory Response

Since embryo implantation depends on a tightly regulated sterile inflammatory response, we next investigated whether the decidual alterations observed in patients with RIF were accompanied by changes in the endometrial inflammatory profile, focusing on the IL-1β pathway.

In the first place, we analysed the mRNA expression of IL-1β, and we did not observe significant modulation in the endometrial biopsies from RIF patients in comparison to samples from fertile women (Figure 4A). However, when we evaluated intracellular IL-1β accumulation by flow cytometry, we evidenced a significant decrease in the frequency of endometrial stromal IL-1β producer cells (vimentin+ IL-1β+) (Figure 4C). The right panels show representative density plots of FACS analysis. Moreover, IL-1β decrease was also accompanied by a reduction in PTGS1 expression (prostaglandin-endoperoxide synthase 1, gene that encodes for the COX1 enzyme), responsible for the production of prostaglandins, in comparison with endometrial samples from fertile women (Figure 4B). These results suggest an inflammatory disruption of the endometrial microenvironment.

### 3.5. The Endometria of RIF Patients Present an Imbalance in the Expression of Angiogenic and ER Stress Markers

Since the decidualization program regulates angiogenesis, we investigated the expression of pro- and anti-angiogenic genes in endometrial samples. As depicted in Figure 5A,B, we observed an increase in the expression of the chemokine with proangiogenic effects, CXCL14, in RIF samples, as well as an increase in arginase 2 (ARG2) expression. Moreover, we found an increase in the soluble fms-like tyrosine kinase-1 (sFlt1), a soluble decoy receptor for VEGFA (Figure 5C) with anti-angiogenic function, and a significant decrease in the expression of its ligand, the main pro-angiogenic factor (Figure 5D).

These results suggest an imbalance in the pro/anti-angiogenic markers that conditions endometria toward an anti-angiogenic response in RIF patients.

Decidualization is accompanied by ER stress and, consequently, the activation of UPR, triggering a sterile inflammatory response through IL-1β production necessary for embryo implantation. Since the IRE1α pathway is involved in the production of IL-1β during decidualization and the induction of angiogenesis [12] and RIF patients displayed a significantly reduced gene expression of these UPR pathways along with a decreased response in the inflammatory and angiogenic processes, we investigated the relevance of the IRE1α pathway in embryo implantation in RIF patients. Based on the integrated bioinformatic analysis of endometrial biopsy arrays, we focused on the genes involved in the processes of ER stress and angiogenesis, and we found that IRE1α is decreased in the endometria of RIF patients in comparison with fertile women and that IRE1α connects the processes of ER stress and angiogenesis (Figure 5E).

Based on this bioinformatic result, we tested the expression of the IRE1α sensor that triggers ER stress and also the main downstream mediators involved in the UPR. As depicted in Figure 5F, the ER stress sensor, IRE1α, displayed a significantly reduced expression in biopsies from RIF patients in comparison to fertile samples. In fact, the expression of the downstream mediators of the IRE1α pathway, TXNIP (thioredoxin-interacting protein) and sXBP1 (the short and functional form of XBP1), was also significantly decreased in RIF samples (Figure 5G,H).

On the other hand, as it has been reported that the production of IL-1β requires the activation of the inflammasome to be released in its active form [27,28], we explored the expression of NLRP3, a critical component of the inflammasome. The NLR family pyrin domain containing 3 (NLRP3) is a key intracellular pattern-recognition receptor responsible for cleaving pro-IL-1β to its active form, IL-1β. In concordance with the reduced expression of IL-1β production and IRE1α pathway mediators in EnSC from RIF patients, endometrial samples from RIF patients had reduced expression of NLRP3 in comparison with endometrial biopsies from fertile women (Figure 5I).

These results indicate that endometrial biopsies from RIF patients displayed a reduction in the IRE1α pathway accompanied by a reduction in the expression of inflammatory and angiogenic markers, which may be related to implantation failures.

Finally, we investigated the relevance of the IRE1α pathway in an in vitro model of implantation. For that purpose, we used pharmacological inhibitors of the IRE1α pathway in decidualized stromal cells, and then we tested the trophoblast expansion index as an approximation of those alterations observed in RIF samples. Therefore, blastocyst-like spheroids (BLS) were cultured on a monolayer of decidualized HESC cells previously treated or not with IRE1α inhibitor (STF-083010), and their adhesion and expansion was monitored by microscopy. We observed that decidualized cells treated with STF (Dec STF) displayed a significant decrease in the expansion index in comparison with decidualized cells (Dec), as well as non-decidualized cells (Non-Dec) (Figure 6).

These results indicate that impairment of the IRE1α pathway may affect the receptivity of decidual cells.

### 3.6. Induction of ER Stress Promotes Angiogenesis in RIF Patients’ Endometrium

Taking into account the reduction in the expression of IRE1α pathway in endometrial samples from RIF patients, we next wondered whether pharmacological induction of ER stress could restore the angiogenic secretory capacity of RIF-derived EnSC. To this end, primary EnSC cultures from RIF patients were treated with thapsigargin (Tg, a non-competitive inhibitor of the sarco/endoplasmic reticulum Ca^2+^-ATPase), at a concentration that did not induce cell death (Figure S1A,B). First, we investigated whether EnSC were able to respond to Tg treatment inducing ER stress and UPR. Figure 7A shows an increase in GRP78 (glucose-regulated protein 78), which encodes for BiP, the main chaperone able to detach from the three ER stress sensors, confirming the induction of UPR pathways under ER stress. EnSC treated with Tg also significantly increased IRE1α and sXBP1 expression (Figure 7B,C).

Then, we recovered the conditioned media from Tg-treated and untreated EnSC primary cultures and tested their ability to modulate endothelial cell migration using a HUVEC wound healing assay. As depicted in Figure 7D, we detected an increase in HUVEC migration in those cultures performed in the presence of conditioned media of RIF EnSC treated with Tg, in comparison with conditioned media from non-treated cells.

The present results indicate that EnSC from RIF patients, despite their altered expression profile, are able to respond to the ER stress induction, activating UPR branches including the IRE1α pathway, changing their secretome, and restoring, at least in part, their pro-angiogenic signalling capacity.

## 4. Discussion

Nowadays, despite the availability of advanced embryo selection strategies and endometrial receptivity assessments, a significant proportion of patients still fail to achieve implantation even after transfer of high-quality embryos [29]. Increasing evidence indicates that recurrent implantation failure is strongly associated with abnormalities in endometrial function, particularly defective decidualization and impaired stromal cell programming [30].

Considering that the decidualization program implies not only phenotypical changes in the endometrial stromal cells, but also in their secretory profile, and is associated with the induction of a sterile inflammation and angiogenic response, here, we wondered if RIF patients display alterations in the decidualization program conditioning decidual cells’ functions impacting embryo implantation. We used complementary approaches that included and integrated bioinformatic analysis of arrays from public databases, validation of the results in endometrial biopsies decidualized in vivo from RIF patients and fertile women, and functional assays with primary cultures of EnSC.

We demonstrated that in vivo decidualization of EnSC from RIF patients displays alterations in the physiological inflammatory and angiogenic responses associated with a decrease in the ER stress signalling. In this sense, our findings identify the IRE1α-UPR axis as a potential mechanistic hub required for endometrial receptivity, inflammation, and angiogenesis during embryo implantation. In fact, the impairment of the IRE1α pathway by pharmacological treatment impacts on in vitro decidualized cells’ receptivity. Since RIF patients have decreased expression of the IRE1α pathway, this may explain the less permissive uterine microenvironment.

Our conclusions are based on several pieces of evidence. First, we found that RIF patients displayed a reduced expression in key decidualization markers (*IGFBP1* and *PGR*), supporting the concept that defective in vivo stromal differentiation is a central feature of implantation failure. Importantly, these alterations were accompanied by increased expression of *MUC1* and reduced *ITGA8* expression, suggesting an endometrial environment less permissive to blastocyst adhesion and stable implantation. Furthermore, conditioned media derived from RIF stromal cells impaired trophoblast migration in the blastocyst-like spheroid assay. Although this supports the concept that altered decidual secretory programming might contribute to implantation failure, further studies utilizing primary EnSC from fertile donors are required to confirm this functional impairment.

Here, we also demonstrated that the endometrium of RIF patients displayed alterations in two key inflammatory mediators, the active form of IL-1β and the prostaglandin synthase, needed for implantation. In particular, we observed a decrease in the expression of *PTGS1*, which encodes for the COX1 enzyme, which is the main source of prostaglandin E2 in these cells. This could be explained based on the fact that IL-1β contributes to prostaglandin production and integrin regulation during implantation, therefore being indirect effects of the lack of UPR in RIF patients [31].

Regarding the production of IL-1β, this cytokine is key in the regulation of the inflammatory response in the peri-implantation period [25,32]. In particular, moderate levels of IL-1β have a pro-implantation function, modulating the expression of extracellular matrix remodelling proteins and increasing the expression of integrins in the endometrium, which will later mediate stable blastocyst adhesion [33]. In this sense, integrins are the dominant glycoproteins involved in the adhesion stage of embryo implantation due to their ability to bind to extracellular matrix ligands and reorganize the cytoskeleton to stabilize blastocyst adhesion. The contribution of integrins in the adhesion of the blastocyst to the luminal epithelium has been very well characterized in different animal models [31,34], in the specific case of ITGA8, in the bovine model [35].

Consistent with this, it has been reported that since IL-1β receptors are expressed in first-trimester placenta and decidua and that exogenous IL-1β stimulates trophoblast cell outgrowth, migration and invasion. It has also been reported that IL-1β increases in the placenta at 6–7-week gestation compared with 8–9-week gestation [9]. This evidence might explain the reduction of trophoblast migration we observed here in the presence of EnSC-conditioned media from primary cultures from RIF patients.

Moreover, the production of IL1β requires the assembly of the NLRP3 inflammasome. The activation of the inflammasome must be tightly regulated to prevent excessive inflammation; hence, the activation of the inflammasome is involved in a diverse range of inflammatory diseases [36,37]. In this sense, we observed a decrease in *NLRP3* expression associated with the decrease observed in IL-1β production and in the trophoblast cell migration. Interestingly, beyond its contribution to inflammation, NLRP3 also induces the epithelial–mesenchymal transition of endometrial epithelial cells and promotes embryo adhesion [38]. Importantly, our group has previously demonstrated that the in vitro decidualization is accompanied by an increase in Caspase-1 activity that directly correlates with NLRP3 upregulation [12]. Therefore, given the defective decidualization profile characterizing RIF patients, the concomitant reduction in *NLRP3* and IL-1β suggests that defective inflammasome-associated signalling may contribute to the impaired inflammatory decidual response observed in RIF patients.

A major finding of this study is the identification of an altered angiogenic profile associated with impaired ER stress signalling in RIF endometria. Successful implantation requires extensive vascular adaptation and angiogenesis to support trophoblast invasion and spiral artery remodelling [10]. Here, RIF samples exhibited reduced *VEGFA* expression together with increased *sFlt1* levels, supporting a shift toward an anti-angiogenic microenvironment. These findings were accompanied by a decreased expression of *IRE1α* and its downstream mediator *sXBP1*, suggesting that downregulation of the IRE1α-UPR pathway may underlie the angiogenic imbalance observed in these patients. An interesting point is that the ER stress/UPR pathways are linked with the angiogenic response. In fact, the short and functional form of XBP1, sXBP1, binds to at least two regions of the *VEGFA* promoter, which could induce an angiogenic response [39]. Consistent with these reports, our integrated bioinformatic analyses identified IRE1α as a central node connecting ER stress, inflammation, and angiogenesis pathways in RIF samples. Importantly, the reduction in *sXBP1* expression observed in RIF biopsies may contribute to the decrease in *VEGFA* expression, reinforcing the concept that defective ER stress signalling compromises angiogenic decidual competence.

In an attempt to provide functional evidence that the ER stress/UPR pathways can modulate endometrial receptivity, we utilized a pharmacological approach. The induction of ER stress in primary cultures of EnSC increased the expression of *GRP78*, *IRE1α*, and *sXBP1* and successfully modulated their secretome, restoring endothelial cell migration capacity. Although thapsigargin induces global ER stress rather than selectively targeting IRE1α signalling, these findings provide functional evidence that modulation of the ER stress response may rescue the angiogenic impairment in RIF-derived stromal cells. However, because Tg also activates the PERK and ATF6 arms of the UPR, the observed angiogenic rescue cannot be exclusively attributed to the IRE1α axis. Therefore, while our molecular and bioinformatic data highlight IRE1α, the specific contribution of this single branch to the functional rescue remains a limitation of the present study.

Collectively, our findings support a model in which defective decidualization in RIF patients is associated with impaired activation of the IRE1α-UPR pathway, leading to alterations in inflammatory signalling, inflammasome activation, and angiogenic imbalance. The resulting anti-angiogenic and poorly receptive endometrial microenvironment may ultimately compromise trophoblast migration and embryo implantation.

Some limitations of this study should be acknowledged. First, the sample size available for the functional assays was limited, reflecting the inherent difficulty of obtaining primary endometrial cultures from both well-characterized RIF patients and fertile women with no gynaecological pathologies. Although we observed reproducible trends and consistent functional responses across these individual donors, this relatively small cohort warrants caution, and larger studies are needed to fully confirm the reproducibility of these effects across the broader RIF population. Second, our study groups are inherently confounded by parity; the fertile cohort comprised women with previous normal pregnancies, while RIF patients were nulliparous. As pregnancy induces long-lasting transcriptomic and functional changes in the endometrium, we cannot completely rule out that some observed differences are related to parity status rather than the RIF pathology itself.

Another critical limitation relates to the trophoblast migration assay, which compared conditioned media from RIF-derived EnSC with that of a decidualized immortalized HESC line. While utilizing primary EnSC from fertile controls would be the ideal experimental design to confirm these specific functional defects, the inherent difficulty in obtaining sufficient endometrial tissue from fertile women to yield enough primary cells for extended functional assays necessitated the use of a standardized cell line.

Although our pharmacological approach provides preliminary evidence that ER stress modulation may influence specific features of endometrial receptivity, further studies are required to determine whether these effects operate in vivo. Importantly, our in vitro implantation model utilizing the specific IRE1α endonuclease inhibitor STF-083010 demonstrated a significant decrease in the trophoblast expansion index, providing functional evidence that the IRE1α axis is mechanistically required for proper endometrial receptivity.

## 5. Conclusions

In summary, our integrated bioinformatic and experimental approach supports an association between RIF and the transcriptional dysregulation of decidualization, inflammatory, angiogenic networks, and the IRE1α pathway. These findings provide new insight into the molecular pathways governing endometrial receptivity and suggest that modulation of the ER stress–UPR axis may represent a relevant molecular pathway to be explored in future translational research for recurrent implantation failure.

## Figures and Tables

**Figure 1:**
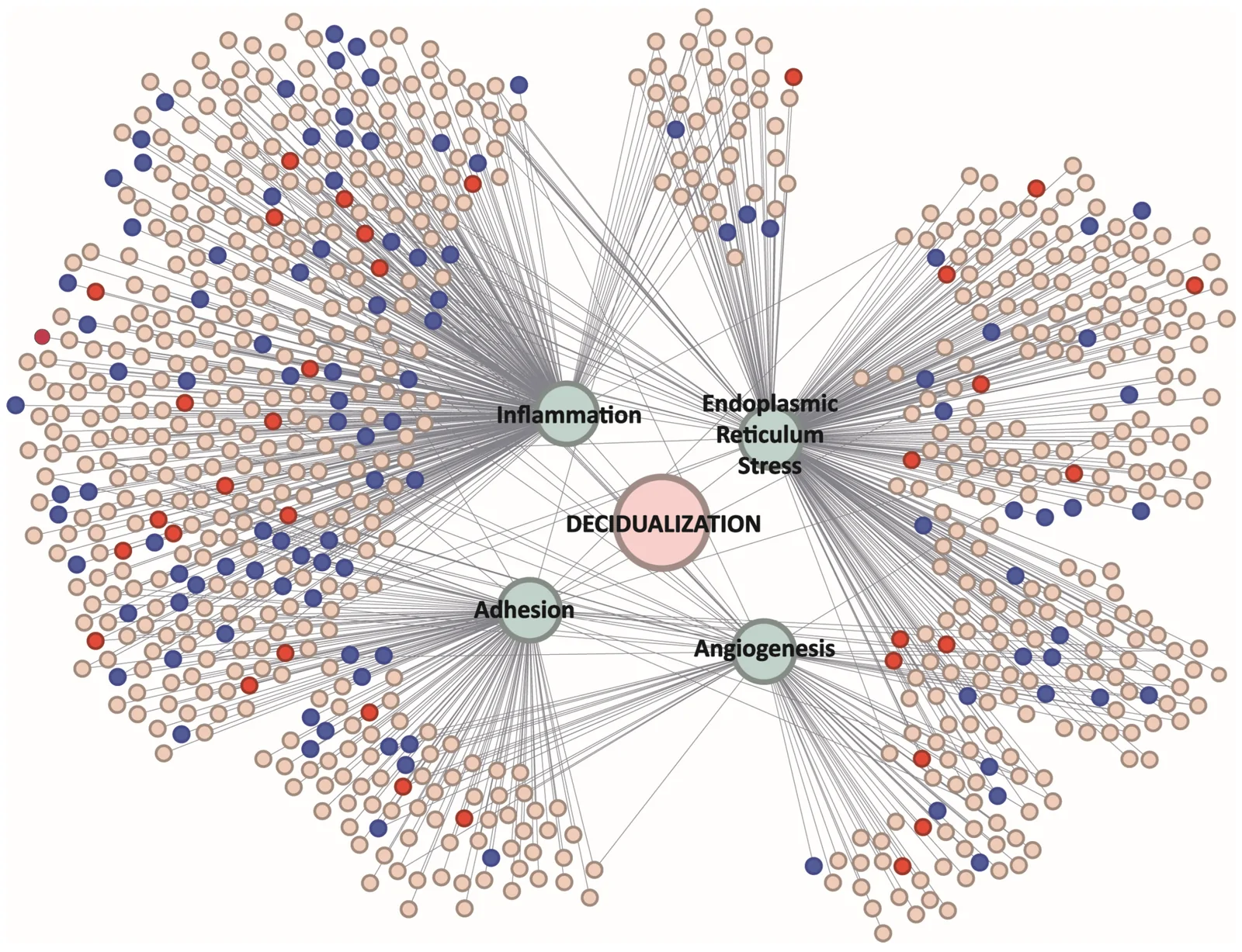
Standardized pathways involved in angiogenesis, ER stress, adhesion, and inflammation were searched in public databases (Reactome, Gene Ontology Biological Process, WikiPathways, and KEGG). Green nodes represent processes; pink nodes represent genes. Gene network was designed with Gephi software. Based on an endometrial array that compares gene expression in endometrial biopsies from RIF or fertile women, we selected those that were modulated. Genes that were significantly upregulated are indicated in red, while those that were downregulated appear in blue.

**Figure 2:**
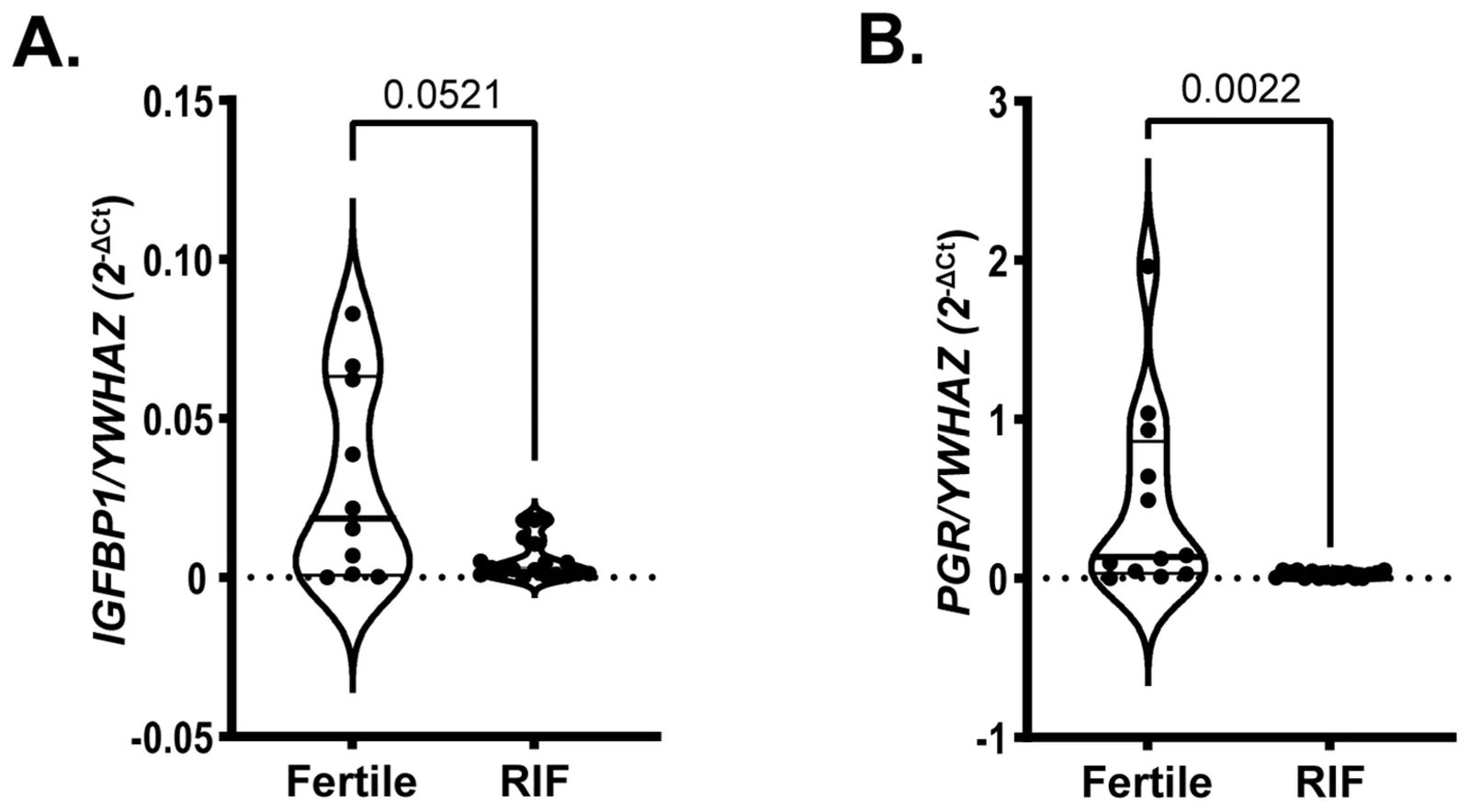
RT-qPCR was performed in endometrial biopsies from fertile and RIF patients, and the expression of different genes was measured as 2^−ΔCt^. (**A**) IGFBP1 (insulin-like growth factor binding protein) and (**B**) PGR (progesterone receptor). Data are displayed by a violin plot, indicating median and quartiles. Mann–Whitney test.

**Figure 3:**
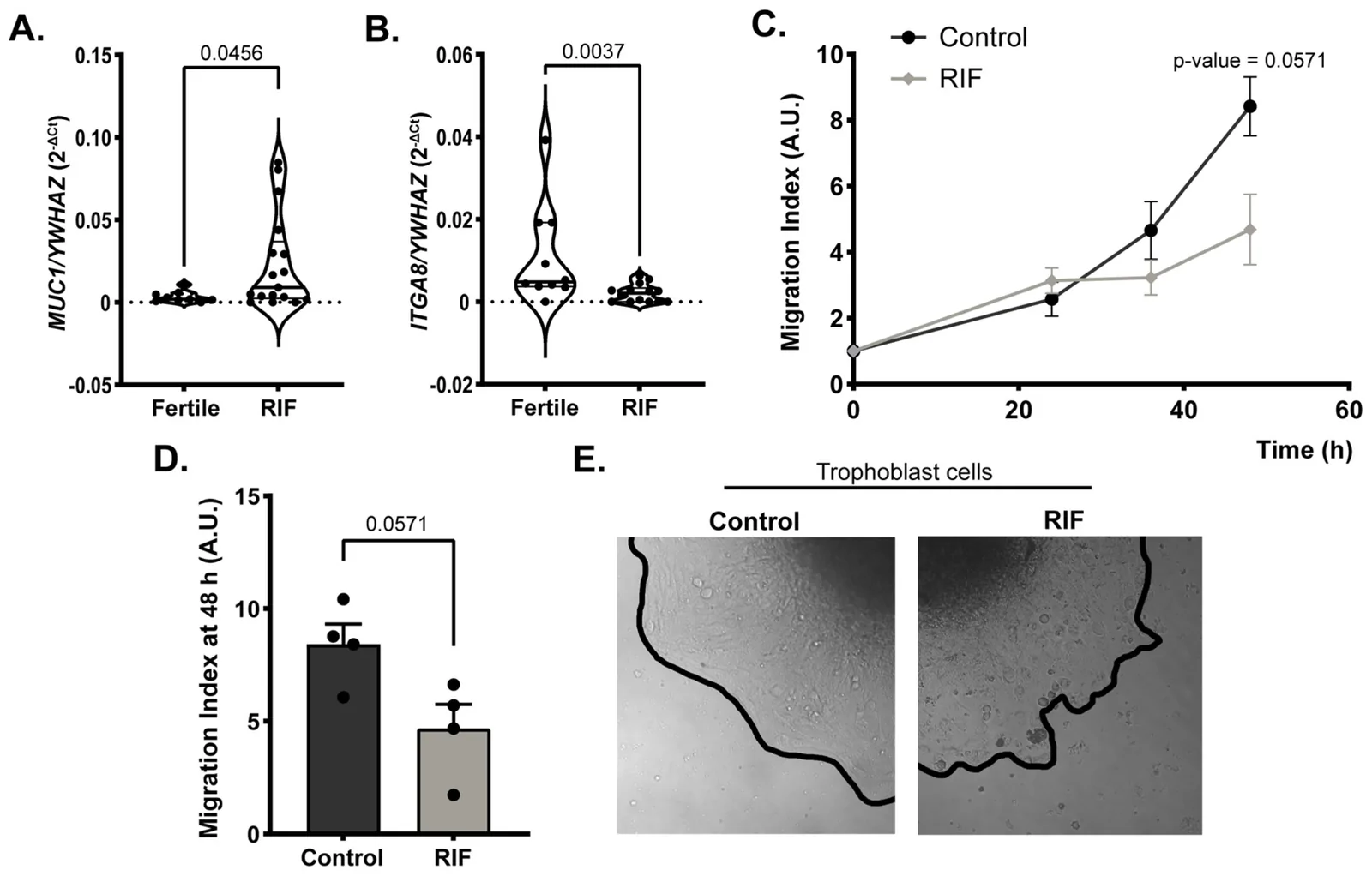
(**A**,**B**) RT-qPCR was performed in endometrial biopsies from fertile and RIF patients, and the expression of different genes was measured as 2^−ΔCt^. (**A**) MUC1 (mucin 1) and (**B**) ITGA8 (integrin subunit alpha 8). Data are displayed by a violin plot, indicating median and quartiles. (**C**) BLS cultured for 48 h with conditioned media from in vitro decidualized HESC (Control) and EnSC isolated from endometrial samples from RIF patients. Migration index of BLS was quantified as total area (spheroid plus outgrown migration area) relative to t0, for all times. Mean ± SEM from n = 4 (independent biological replicates) is shown in (**D**), and representative experiment at 48 h is shown in (**E**) Mann–Whitney test.

**Figure 4:**
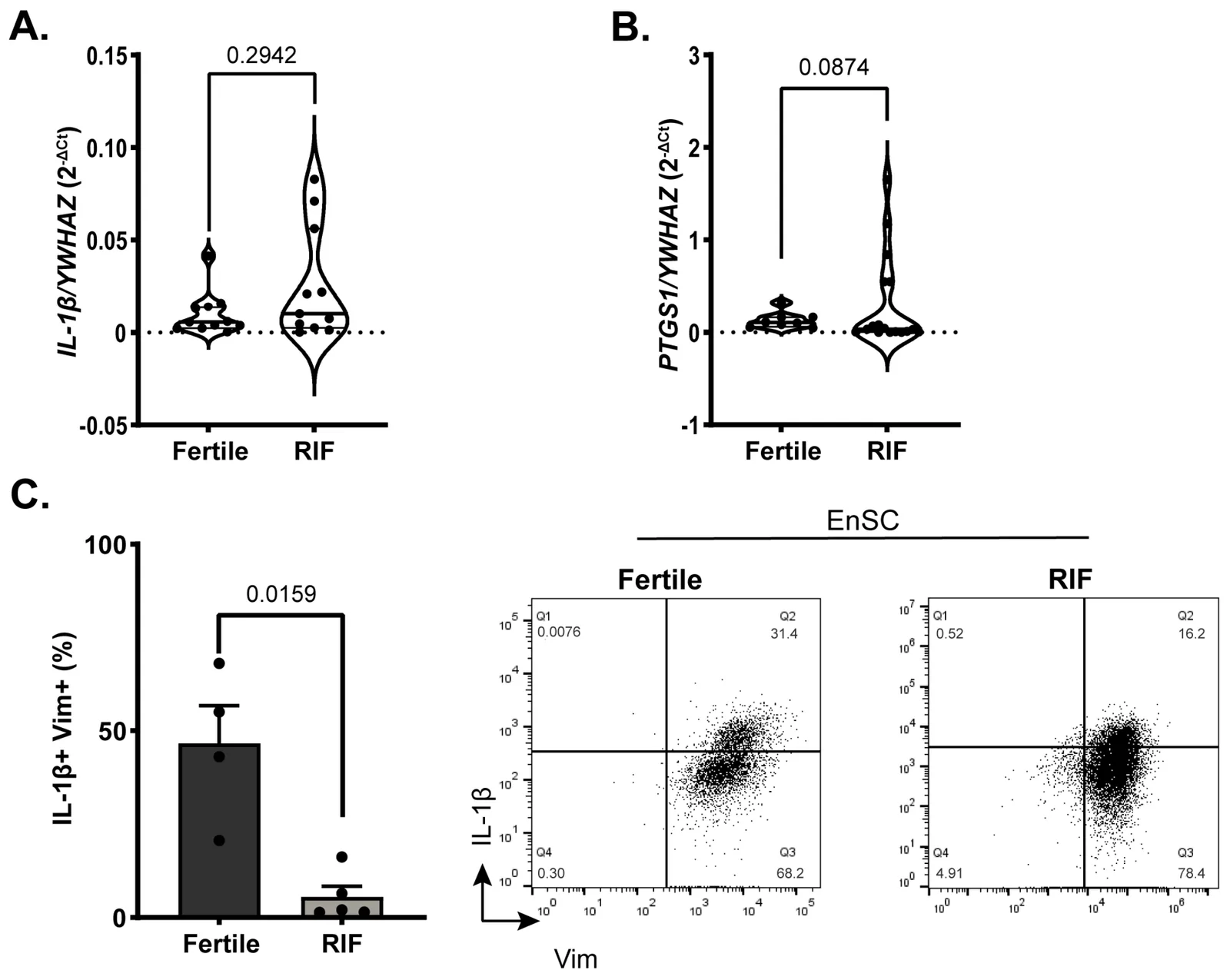
(**A**,**B**) RT-qPCR was performed in endometrial biopsies from fertile women and RIF patients for IL-1β (**A**) and PTGS1 (prostaglandin-endoperoxide synthase 1) (**B**) gene and expressed as 2^−ΔCt^. (**C**) Percentage of IL-1β production of stromal cells isolated from biopsies from fertile women (n = 4) and RIF patients (n = 5) measured by flow cytometry stained with antibodies anti-vimentin and anti-IL1β. Density plot of a representative experiment is shown in right panel. Mann–Whitney test.

**Figure 5:**
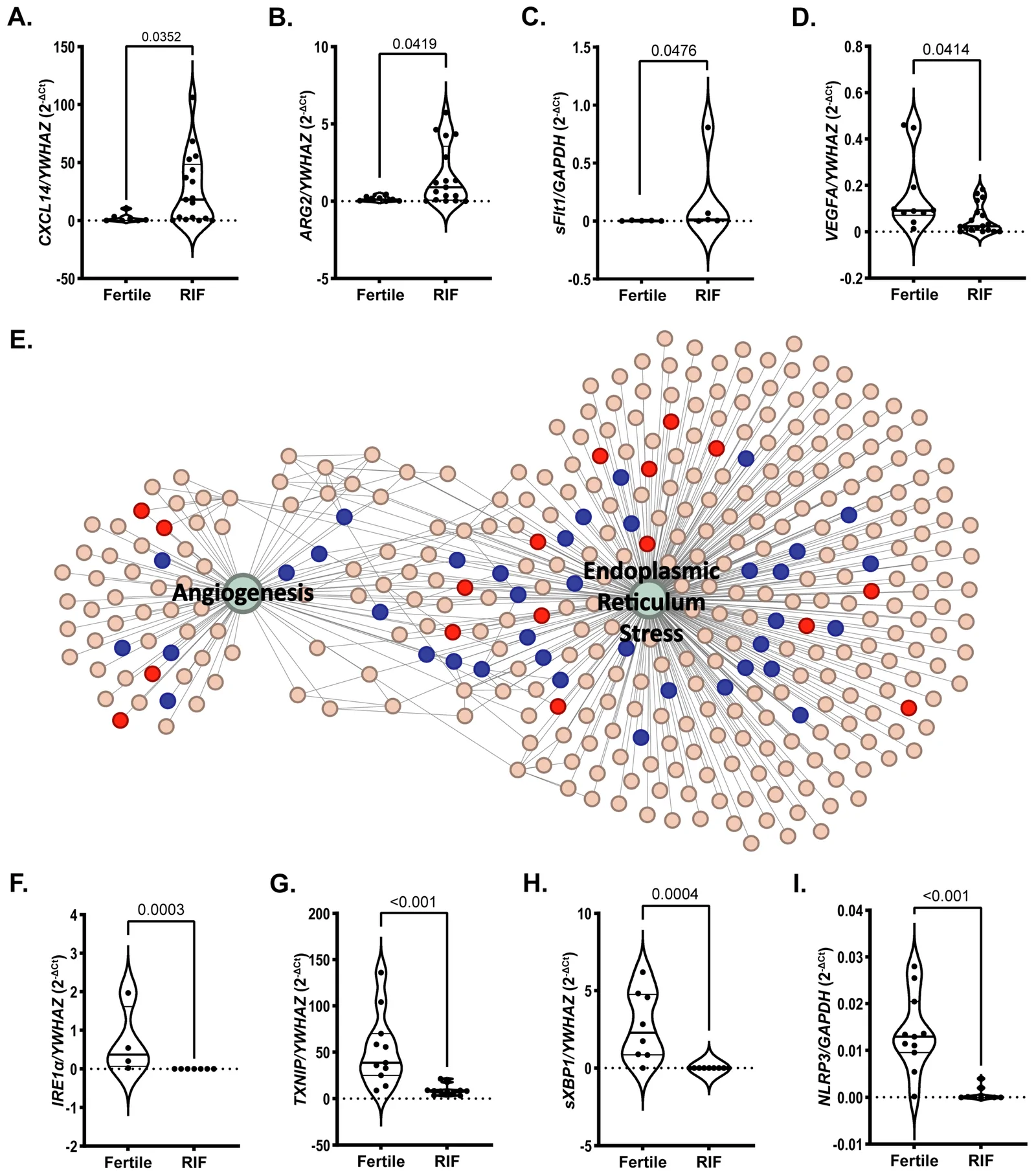
(**A**–**D**) RT-qPCR was performed in endometrial biopsies from fertile and RIF patients, and the expression of different genes was measured as 2^−ΔCt^. (**A**) CXCL14 (chemokine (C-X-C motif) ligand 14), (**B**) ARG2 (arginase 2), (**C**) sFlt1 (soluble fms related receptor tyrosine kinase 1), and (**D**) VEGFA (vascular endothelial growth factor A). Data are displayed by a violin plot, indicating median and quartiles. Mann–Whitney test. (**E**) Standardized pathways involved in senescence and ER stress were searched in public databases (Reactome, Gene Ontology Biological Process, WikiPathways, and KEGG). Green nodes represent processes; pink nodes represent genes. Gene network was designed with Gephi software. Based on an endometrial array that compares gene expression in endometrial biopsies from RIF or fertile women, we selected those that were modulated. Genes that were significantly upregulated are indicated in red, while those that were downregulated appear in blue. (**F**–**I**) RT-qPCR was performed in endometrial biopsies from fertile and RIF patients, and the expression of different genes was measured as 2^−ΔCt^. Arbitrary units are shown. IRE1α: inositol-requiring enzyme 1α, sXBP1: spliced X-box binding protein 1, NLRP3: NLR family pyrin domain containing 3. Mann–Whitney test.

**Figure 6:**
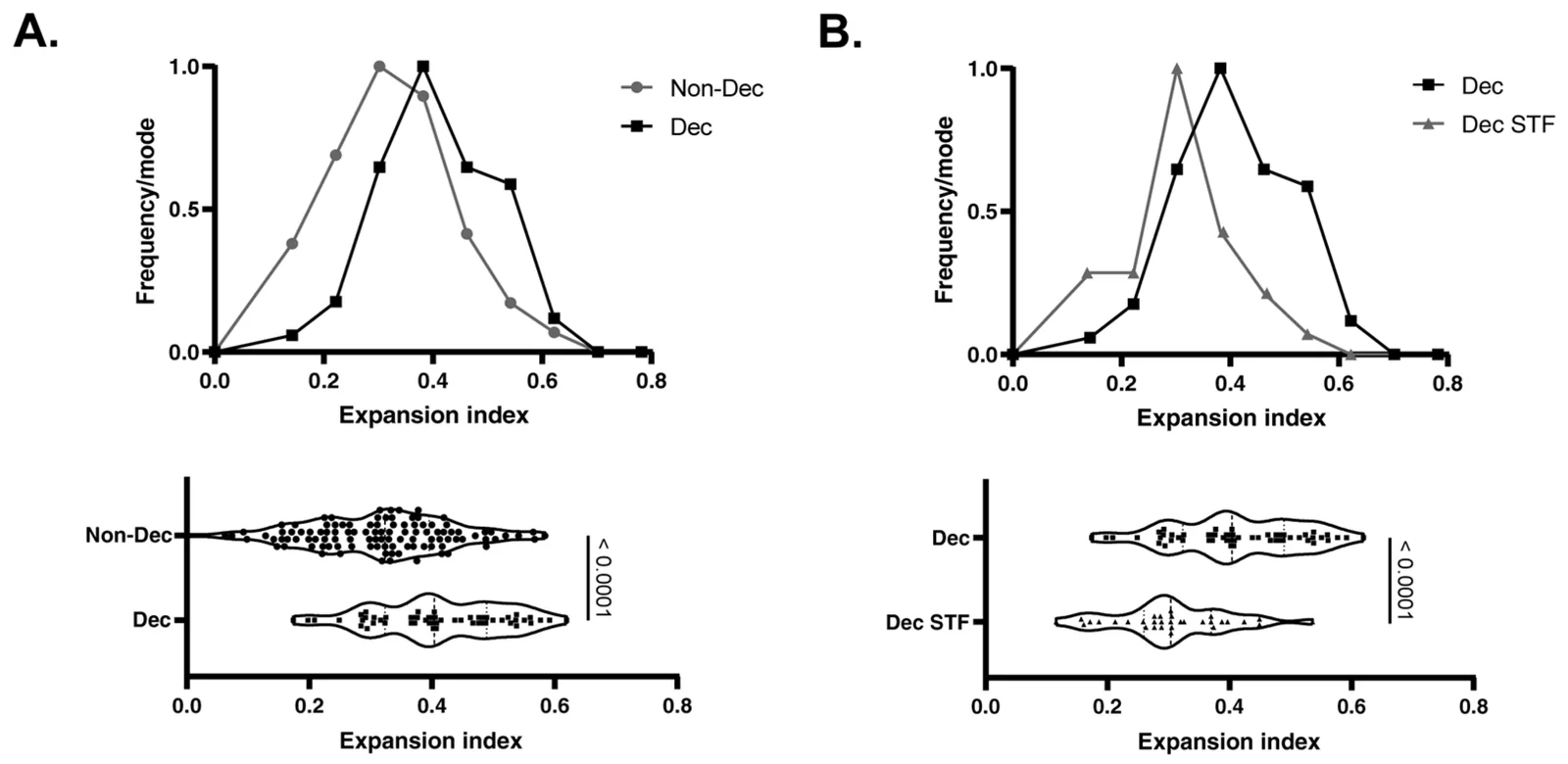
(**A**) BLS co-cultivated with decidualized (Dec) or non-decidualized (Non-Dec) HESC. (**B**) Dec cells were treated with ER stress inhibitor STF083 (Dec STF). Upper panels show histograms showing BLS spreading, and lower panels show violin plots, where each dot represents a BLS. *t*-test.

**Figure 7:**
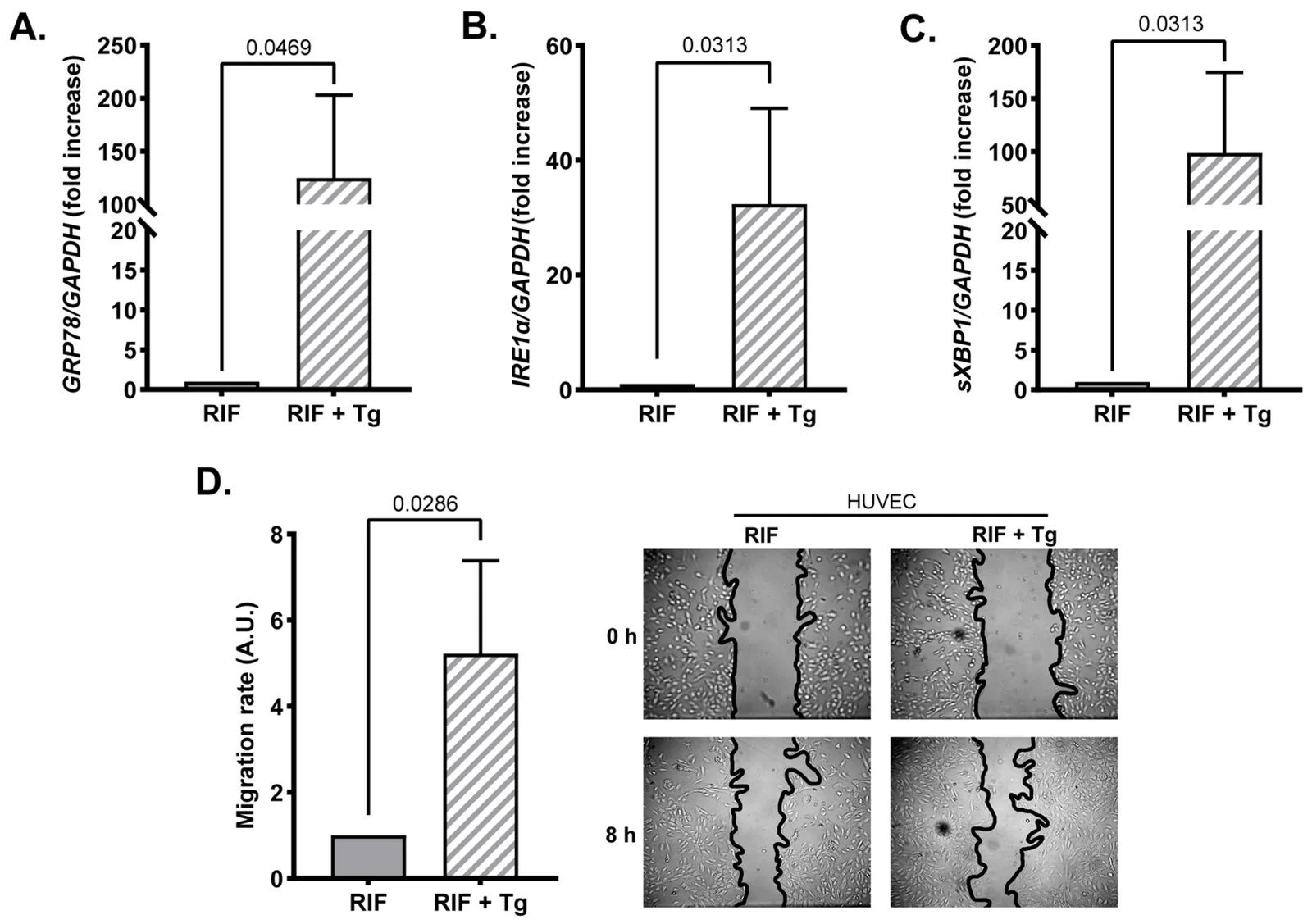
(**A**–**C**) RT-qPCR was performed in EnSC isolated from endometrial biopsies from RIF patients previously treated with Tg or not (n = 6). The expression of different genes is expressed as fold increase. GRP78: glucose-regulated protein 78. (**D**) Migration rate of HUVEC cultivated with conditioned media of EnSC from endometrial samples from RIF patients treated with Tg (RIF + Tg) or not treated (RIF) (n = 3, independent biological replicates). Mean ± SEM, Wilcoxon test.

**Table 1:** Patients’ demographic characteristics (median and range).

	Fertile Women	RIF Patients
N	12	20
Age	41 (32–46)	38 (33–46)
IMC	27.45 (19.49–34.68)	22.59 (17.42–26.91)
Births	2 (2–5)	0
IVF failures	0	3 (2–9)

**Table 2:** Primer sequences of genes evaluated by RT-qPCR. Oligonucleotide primers were designed using the online tool Primer Blast (NCBI NIH, Bethesda, MD, USA).

Gene	Primer Fw (5′-3′)	Primer Rv (5′-3′)
*TXNIP*	GACCGCCCGAGCCAGCCAAC	AGAGACAGACACCCGCCCATCAGG
*ARG2*	TCAGTGCTGCGGATCATGT	CACTCCTTTTCTTTTCTGCCCTT
*sXBP1*	CTGAGTCCGCAGCAGGTG	GGAGGCTGGTAAGGAACTGG
*CXCL14*	TGAAGCCAAAGTACCCGCAC	CCCTATTCTTCGTAGACCCTGC
*GRP78*	ATCAACGAGCCTACGGCAG	AGACACATCGAAGGTTCCGC
*GAPDH*	ATCACCATCTTCCAGGAGCGA	GGTTCACACCCATGACGAAC
*IGFBP1*	GAGCCCTGCCGAATAGAACT	GATGTCTCACACTGTCTGCTG
*IL-1β*	GGATATGGAGCAACAAGTGGTGT	CACGCAGGACAGGTACAGATT
*IRE1a*	GGGTCTGAGGAAGGTGATGC	AACATACAGAGTGGGCGTCAG
*ITGA8*	TGGGAGTTTCTACTGGCAAGG	GCAGCAACTGAGTATCCAAGGT
*MUC1*	CCTCACAGTGCTTACAGTTGTT	AAGAGACCCCAGTAGACAAAGC
*NLRP3*	AAAGGAAGTGGACTGCGAGA	TTCAAACGACTCCCTGGAAC
*PGR*	GGTCTACCCGCCCTATCTCA	GTAGTTGTGCTGCCCTTCCA
*PRL*	TCATCTGGTCACGGAAGTACG	GTTTCAGGATGAACCTGGCTG
*PTGS1*	GATGCCTTCTCTCGCCAGATT	TGCCATCTCCTTCTCTCCTACG
*sFlt1*	AGAGGTGAGCACTGCAACAA	TCTCCTCCGAGCCTGAAAGT
*VEGFA*	CGCAGCTACTGCCATCCAAT	GTGAGGTTTGATCCGCATAATCT
*YWHAZ*	CAGAGAGAAAATTGAGACGGAGC	CAGAGAGAAAATTGAGACGGAGC

## Data Availability

Used transcriptomic data is publicly available at GEO (https://www.ncbi.nlm.nih.gov/geo/ accessed on 16 March 2026) under accessions: GSE234354, GSE92324.

## Supplementary Materials

The following supporting information can be downloaded at: https://www.mdpi.com/article/10.3390/biology15191693/s1, Table S1. List of genes modulated in processes associated with embryo implantation in RIF patients. Figure S1. Cellular viability after treatment with Tg.
