# Supplementary material for: Altered IRE1α-Dependent Decidual Programming Links Impaired Inflammation and Angiogenesis in Recurrent Implantation Failure
Source: Biology (Basel). 2026 Sep 23;15(19):1693. doi: 10.3390/biology15191693 (PMC13663388; doi:10.3390/biology15191693)
Supplement: Supplementary file 1 [file biology-15-01693-s001.zip › biology-4566633-supplementary.pdf]

**Table S1. List of genes modulated in processes associated with embryo implantation in RIF patients.** Standardized pathways involved in angiogenesis, ER stress, adhesion and inflammation were searched in public databases (Reactome, Gene Ontology Biological Process, WikiPathways, KEGG). Based on an endometrial array that compares gene expression in endometrial biopsies from RIF or fertile women, we selected those that were modulated (Downregulated or Upregulated).

| Gene | Modulation | Processes |
|---|---|---|
| ANG | Downregulated | Endoplasmic Reticulum Stress, Adhesion |
| LAMB3 | Downregulated | Adhesion |
| MMP7 | Downregulated | Adhesion |
| FGR | Downregulated | Inflammation, Adhesion |
| RAC2 | Downregulated | Adhesion |
| ITGA3 | Downregulated | Adhesion |
| MAP3K5 | Downregulated | Endoplasmic Reticulum Stress, Angiogenesis |
| CDKN2B | Downregulated | Endoplasmic Reticulum Stress |
| ITGAX | Downregulated | Inflammation, Adhesion |
| SORBS1 | Downregulated | Adhesion |
| GPX3 | Downregulated | Endoplasmic Reticulum Stress, Angiogenesis |
| COMP | Downregulated | Endoplasmic Reticulum Stress, Adhesion |
| MADCAM1 | Downregulated | Inflammation, Adhesion |
| VCAM1 | Downregulated | Inflammation, Adhesion |
| ADAMTS16 | Downregulated | Adhesion |
| NOS3 | Downregulated | Endoplasmic Reticulum Stress, Angiogenesis |
| ITGB3 | Downregulated | Angiogenesis, Adhesion |
| TRIB3 | Downregulated | Endoplasmic Reticulum Stress, Angiogenesis |
| KIR2DL4 | Downregulated | Endoplasmic Reticulum Stress, Inflammation |
| CAPN9 | Downregulated | Adhesion |
| LCN2 | Downregulated | Endoplasmic Reticulum Stress, Inflammation |
| NUPR1 | Downregulated | Endoplasmic Reticulum Stress, Inflammation |
| CDH3 | Downregulated | Adhesion |
| CLDN10 | Downregulated | Adhesion |
| VNN1 | Downregulated | Endoplasmic Reticulum Stress, Inflammation |
| FGA | Downregulated | Adhesion |
| TIMP3 | Downregulated | Angiogenesis, Adhesion |
| VAV3 | Downregulated | Endoplasmic Reticulum Stress, Angiogenesis, Adhesion |
| THBS2 | Downregulated | Angiogenesis, Adhesion |
| FGB | Downregulated | Adhesion |
| ITGA2 | Downregulated | Adhesion |

| | | |
|---|---|---|
| COL17A1 | Downregulated | Inflammation, Adhesion |
| AQP3 | Downregulated | Endoplasmic Reticulum Stress |
| BATF3 | Downregulated | Endoplasmic Reticulum Stress |
| ITGAD | Downregulated | Adhesion |
| AGT | Downregulated | Inflammation |
| BCL2L10 | Downregulated | Endoplasmic Reticulum Stress |
| BMP6 | Downregulated | Inflammation |
| C2CD4A | Downregulated | Inflammation |
| C3 | Downregulated | Inflammation |
| CCL14 | Downregulated | Inflammation |
| CCL23 | Downregulated | Inflammation |
| CCR1 | Downregulated | Inflammation |
| CD247 | Downregulated | Inflammation |
| CD300C | Downregulated | Inflammation |
| CD38 | Downregulated | Endoplasmic Reticulum Stress |
| CD68 | Downregulated | Inflammation |
| CXCL14 | Downregulated | Inflammation |
| CXCR3 | Downregulated | Inflammation |
| CXCR4 | Downregulated | Inflammation |
| EDNRB | Downregulated | Inflammation |
| EIF4E3 | Downregulated | Inflammation |
| ELF3 | Downregulated | Inflammation |
| ERN1 | Downregulated | Angiogenesis, Endoplasmic Reticulum Stress |
| ERP27 | Downregulated | Endoplasmic Reticulum Stress |
| F13A1 | Downregulated | Inflammation |
| FAM3C | Downregulated | Inflammation |
| FASLG | Downregulated | Inflammation |
| FCER1G | Downregulated | Inflammation |
| FCGR1A | Downregulated | Inflammation |
| FLT3 | Downregulated | Inflammation |
| FXYD2 | Downregulated | Endoplasmic Reticulum Stress |
| GADD45A | Downregulated | Endoplasmic Reticulum Stress |
| GATA3 | Downregulated | Inflammation |
| GPX5 | Downregulated | Endoplasmic Reticulum Stress |
| GRB7 | Downregulated | Angiogenesis |
| GTSE1 | Downregulated | Endoplasmic Reticulum Stress |
| GZMA | Downregulated | Inflammation |
| HAS2 | Downregulated | Endoplasmic Reticulum Stress |
| HAVCR2 | Downregulated | Inflammation |
| IDO1 | Downregulated | Inflammation |
| IL2RB | Downregulated | Inflammation |
| IL6ST | Downregulated | Inflammation |
| INHBB | Downregulated | Endoplasmic Reticulum Stress |

| | | |
|---|---|---|
| KCNK2 | Downregulated | Endoplasmic Reticulum Stress |
| KIR2DL1 | Downregulated | Inflammation |
| KIR2DL3 | Downregulated | Inflammation |
| KIR3DL1 | Downregulated | Inflammation |
| KIR3DL2 | Downregulated | Inflammation |
| KLRC1 | Downregulated | Inflammation |
| KLRD1 | Downregulated | Inflammation |
| LBP | Downregulated | Inflammation |
| LCP1 | Downregulated | Inflammation |
| LIF | Downregulated | Inflammation |
| MMRN1 | Downregulated | Angiogenesis |
| NCAM1 | Downregulated | Inflammation |
| NCR3 | Downregulated | Inflammation |
| NFE2 | Downregulated | Endoplasmic Reticulum Stress |
| NFKBIZ | Downregulated | Inflammation |
| NKG7 | Downregulated | Inflammation |
| OASL | Downregulated | Inflammation |
| ORM2 | Downregulated | Inflammation |
| OSMR | Downregulated | Inflammation |
| PLA2G2A | Downregulated | Inflammation |
| PPARGC1A | Downregulated | Endoplasmic Reticulum Stress |
| PSTPIP1 | Downregulated | Inflammation |
| S100A9 | Downregulated | Inflammation |
| SLA | Downregulated | Inflammation |
| SLC1A1 | Downregulated | Endoplasmic Reticulum Stress |
| SOCS3 | Downregulated | Inflammation |
| STAP1 | Downregulated | Inflammation |
| TEX15 | Downregulated | Endoplasmic Reticulum Stress |
| TGFB2 | Downregulated | Endoplasmic Reticulum Stress |
| TGM2 | Downregulated | Angiogenesis |
| TIFAB | Downregulated | Endoplasmic Reticulum Stress |
| TUBA4A | Downregulated | Inflammation |
| UGT1A6 | Downregulated | Angiogenesis |
| XCL1 | Downregulated | Inflammation |
| ZAP70 | Downregulated | Inflammation |
| ADAMTS8 | Upregulated | Adhesion |
| BMPR1B | Upregulated | Inflammation |
| CCL25 | Upregulated | Inflammation |
| CHAF1B | Upregulated | Endoplasmic Reticulum Stress |
| CHST4 | Upregulated | Inflammation |
| CLIC3 | Upregulated | Angiogenesis |
| COL6A6 | Upregulated | Angiogenesis, Adhesion |
| COL9A2 | Upregulated | Angiogenesis, Adhesion |

| | | |
|---|---|---|
| DUOX1 | Upregulated | Endoplasmic Reticulum Stress |
| DUOXA1 | Upregulated | Inflammation |
| ENPP3 | Upregulated | Inflammation |
| FBN3 | Upregulated | Adhesion |
| FBP1 | Upregulated | Endoplasmic Reticulum Stress |
| FOLR1 | Upregulated | Endoplasmic Reticulum Stress |
| GPX2 | Upregulated | Endoplasmic Reticulum Stress |
| IFNGR1 | Upregulated | Inflammation |
| ITGB8 | Upregulated | Adhesion |
| KCNK13 | Upregulated | Inflammation |
| MIR657 | Upregulated | Inflammation |
| MSX1 | Upregulated | Endoplasmic Reticulum Stress |
| NLRP5 | Upregulated | Inflammation |
| OSM | Upregulated | Inflammation |
| PDK4 | Upregulated | Endoplasmic Reticulum Stress |
| PRKAG2 | Upregulated | Endoplasmic Reticulum Stress |
| PRKCQ | Upregulated | Inflammation |
| PTGS1 | Upregulated | Endoplasmic Reticulum Stress |
| PTGS2 | Upregulated | Inflammation, Angiogenesis, Endoplasmic Reticulum Stress |
| SCUBE1 | Upregulated | Inflammation, Adhesion |
| SDK2 | Upregulated | Adhesion |
| SFRP2 | Upregulated | Endoplasmic Reticulum Stress |
| SLC34A1 | Upregulated | Endoplasmic Reticulum Stress |
| SPHK1 | Upregulated | Inflammation, Angiogenesis, Endoplasmic Reticulum Stress |
| TLR5 | Upregulated | Inflammation |
| ULBP1 | Upregulated | Inflammation |
| XDH | Upregulated | Angiogenesis |

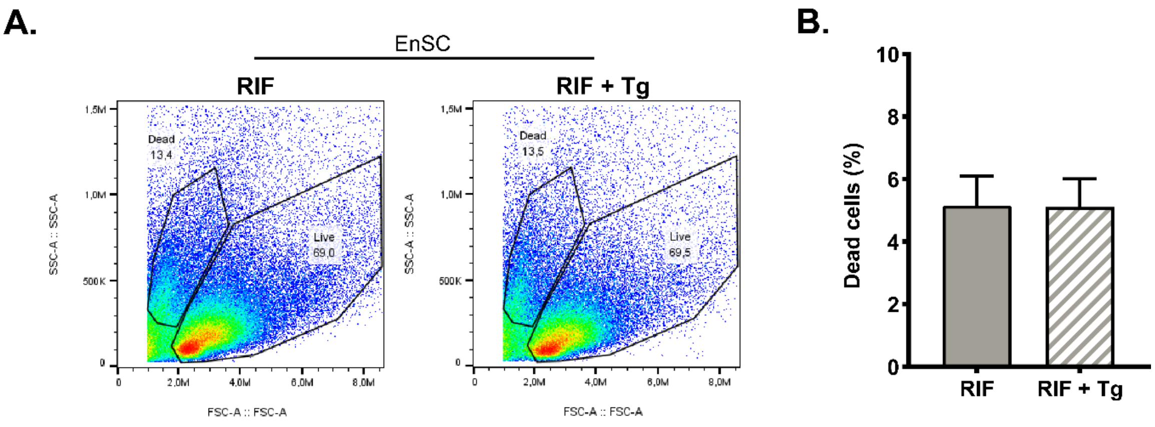


**Figure S1. Cellular viability after treatment with Tg.** (**A**) Representative dot plot of

EnSC isolated from biopsies from RIF patients treated or not with Tg, gates showing dead and live cells. (**B**) Percentage of dead cells of EnSC isolated from biopsies from RIF patients treated or not with Tg (n = 5). Mean ± SEM, Wilcoxon test.
